# RICTOR amplification identifies a subgroup in small cell lung cancer and predicts response to drugs targeting mTOR

**DOI:** 10.18632/oncotarget.13362

**Published:** 2016-11-15

**Authors:** Nneha Sakre, Gary Wildey, Mohadese Behtaj, Adam Kresak, Michael Yang, Pingfu Fu, Afshin Dowlati

**Affiliations:** ^1^ Case Comprehensive Cancer Center, Case Western Reserve University, Cleveland, Ohio, 44106 USA; ^2^ Division of Hematology and Oncology, University Hospitals Cleveland Medical Center, Cleveland, Ohio, 44106 USA; ^3^ Department of Pathology, University Hospitals Cleveland Medical Center, Cleveland, Ohio, 44106 USA; ^4^ Epidemiology and Biostatistics, Case Western Reserve University, Cleveland, Ohio, 44106 USA

**Keywords:** RICTOR, mTORC1/2 inhibitors, CNV, amplification, small cell lung cancer

## Abstract

Small cell lung cancer (SCLC) is an aggressive cancer that represents ~15% of all lung cancers. Currently there are no targeted therapies to treat SCLC. Our genomic analysis of a metastatic SCLC cohort identified recurrent *RICTOR* amplification. Here, we examine the translational potential of this observation. *RICTOR* was the most frequently amplified gene observed (~14% patients), and co-amplified with *FGF10* and *IL7R* on chromosome 5p13. *RICTOR* copy number variation correlated with RICTOR protein expression in SCLC cells. In parallel, cells with *RICTOR* copy number (CN) gain showed increased sensitivity to three mTOR inhibitors, AZD8055, AZD2014 and INK128 in cell growth assays, with AZD2014 demonstrating the best inhibition of downstream signaling. SCLC cells with *RICTOR* CN gain also migrated more rapidly in chemotaxis and scratch wound assays and were again more sensitive to mTOR inhibitors. The overall survival in SCLC patients with *RICTOR* amplification was significantly decreased (p = 0.021). Taken together, our results suggest that SCLC patients with *RICTOR* amplification may constitute a clinically important subgroup because of their potential response to mTORC1/2 inhibitors.

## INTRODUCTION

Lung cancer is divided into two disease types: small cell lung cancer (SCLC) and non-small cell lung cancer (NSCLC), based upon the histologic appearance of the cancer cells and the behavior of the disease. SCLC makes up about 15 percent of all lung cancers and is the subtype most closely associated with cigarette smoking [1]. SCLC is an aggressive cancer that tends to grow and spread quickly. The aggressive and distant spread of SCLC led the Veterans Administration Lung Study Group in 1957 to create a dichotomized staging system termed limited stage (LS), characterized by a tumor volume encompassed in one radiation portal, and extensive stage (ES), encompassing all forms of metastatic disease [2]. Because most SCLC patients present with metastatic disease, treatment remains platinum-based chemotherapy and surgery is rarely performed (<3% of cases). Currently there are no molecular targeted approaches to treat SCLC.

In this regard, two whole exome studies by Rudin et al [3] and Peifer et al [4] analyzed 40 and 27 SCLC tumors, respectively. These studies identified only two mutated genes that are readily actionable, *PTEN* (phosphatase and tensin homolog), mutated in 10% of patients and *FGFR1* (fibroblast growth factor receptor 1), amplified in 6% of patients. A more recent whole genome study by George et al [5] found a similar low mutation frequency in targetable genes, such as *KIT* (KIT proto-oncogene receptor tyrosine kinase) (6%) and *PIK3CA* (phosphatidylinositol-4,5-biphosphate 3-kinase catalytic subunit alpha) (3%). While groundbreaking, there are two limitations of these studies. First, the great majority of tumors analyzed were from a rare subgroup of patients with early stage, resectable disease (LS) and second, none of these studies provided clinical correlates, such as response to therapy or survival, with gene alterations.

Therefore, we recently completed a targeted exome sequencing analysis of ES SCLC tumors and correlated gene mutations with clinical outcome [6]. In this study we identified *RICTOR* (RPTOR independent companion of MTOR, complex 2) amplification as one of the most frequent, actionable gene alterations in SCLC. RICTOR is a subunit of mTORC2 (mammalian target of rapamycin, complex 2) and regulates cell growth in response to hormonal, but not nutrient, stimulation via phosphorylation of AKT1 (AKT serine/threonine kinase 1), SGK1 (serum/glucocorticoid regulated kinase 1) and PKCα (protein kinase Cα) [7]. mTORC2 also has been shown to regulate epithelial mesenchymal transition (EMT) [8] as well as invasion and metastasis in colon and breast cancer [9, 10]. Through these combined actions, mTORC2 is thought to play an essential role in the aggressive growth and motility characteristics of cancer.

Here we have built upon our initial finding of *RICTOR* amplification in SCLC in order to determine its clinical importance. We provide clinical correlates of *RICTOR* amplification in SCLC, and have used model SCLC cell lines with various levels of *RICTOR* copy number (CN) gain in order to analyze its downstream effects on cell growth and migration. Finally, we have used mTOR inhibitors currently under investigation in clinical trials to examine the translational potential of RICTOR inhibition in SCLC patients.

## RESULTS

### *RICTOR* is frequently amplified in SCLC tumors

**Figure 1:**
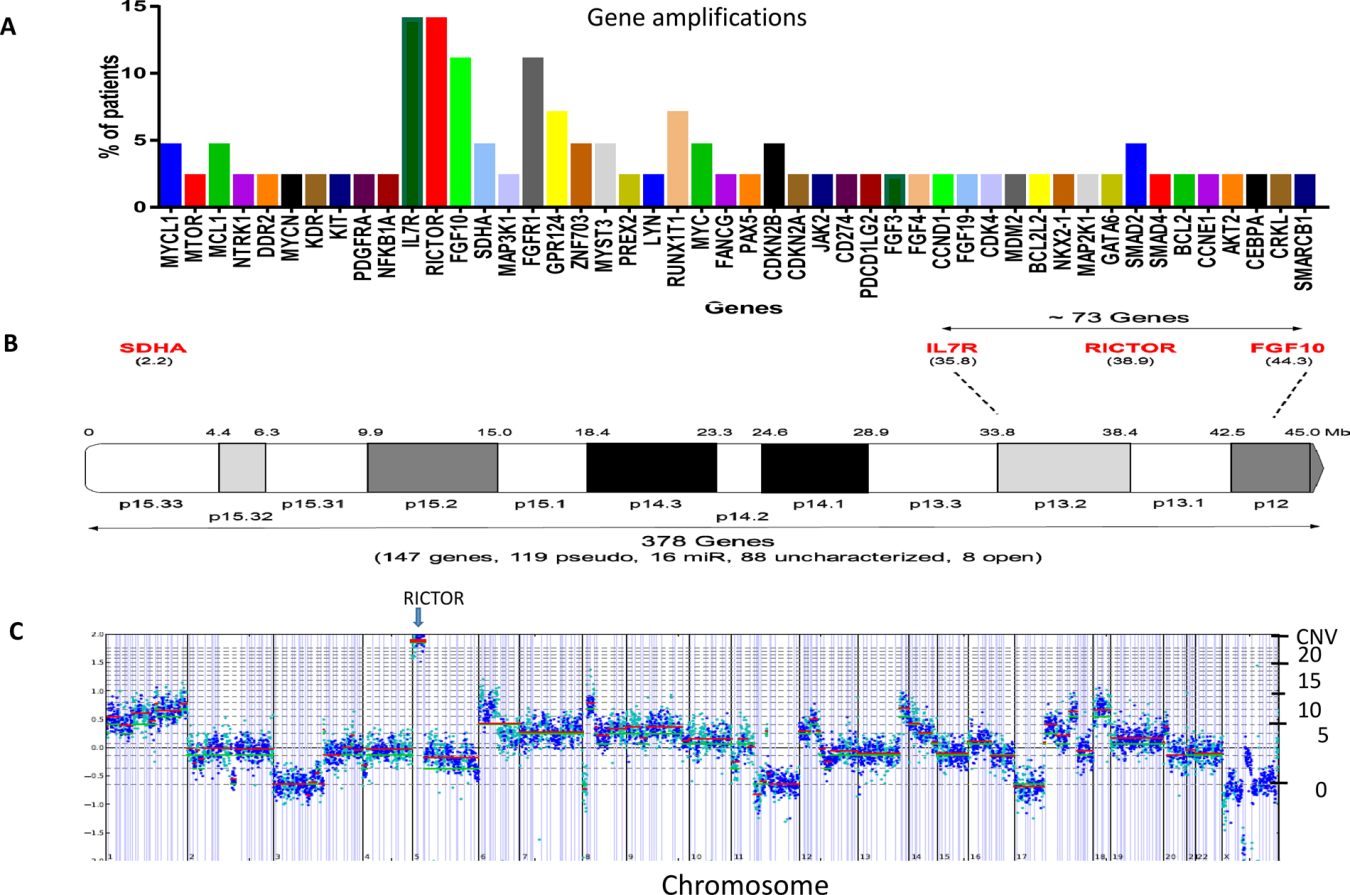
RICTOR amplification in SCLC patients **A**. Amplification profile, arranged by chromosomal location, of SCLC cohort. **B**. Localization of genes on chromosome 5p. **C**. CNV plot of a SCLC tumor sample with amplification of the *RICTOR* gene at 20 copies on 5p13. The chromosomal location is provided on the X-axis and the gene copy number on the Y-axis. The *RICTOR* gene is more frequently amplified than either *FGFR1* or *MYC*.

Targeted exome sequencing data was used to derive chromosomal amplifications [11]. The most frequent amplified genes observed (~14% patients) were *RICTOR*, *FGF10* (fibroblast growth factor 10) and *IL7R* (interleukin 7 receptor), all of which are localized to chromosome 5p13 (Figure 1A). Another gene, *SDHA* (succinate dehydrogenase complex flavoprotein subunit A), located on 5p15, was less frequently amplified (~5%), while several genes located on the 5q arm were not amplified [*CSF1R* (colony stimulating factor 1 receptor) (q32), *PDGFRB* (platelet derived growth factor receptor beta) (q33.1), *FLT4* (fms related tyrosine kinase 4) (q35.3), *RAD50* (RAD50 double strand break repair protein) (q31)]. The detailed localization of genes on chromosome 5p13 is shown in Figure 1B. *RICTOR* amplification ranged from a CNV of 6 to 20; a plot of tumor copy number variation (CNV) demonstrating high, focal *RICTOR* amplification is shown in Figure 1C (see Supplementary Figure 1 for additional plots). *FGFR1*, the second most frequently amplified gene in our cohort (10%), also co-amplified with several neighboring genes located on chromosome 8p11, including *GPR124* (G protein coupled receptor 124)*, ZNF703* (zinc finger protein 703) and *MYST3* (KAT6A, lysine acetyltransferase 6A). Interestingly, *MYC* (v-myc avian myelocytomatosis viral oncogene homolog) family amplification was infrequent. Supplementary Table 1 depicts the distribution of amplified genes among the 21 patients with at least one gene amplification. Because of its potential as a novel therapeutic target in SCLC, we focused on *RICTOR* amplification in all subsequent studies.

### SCLC cell lines as models for RICTOR amplification

**Figure 2:**
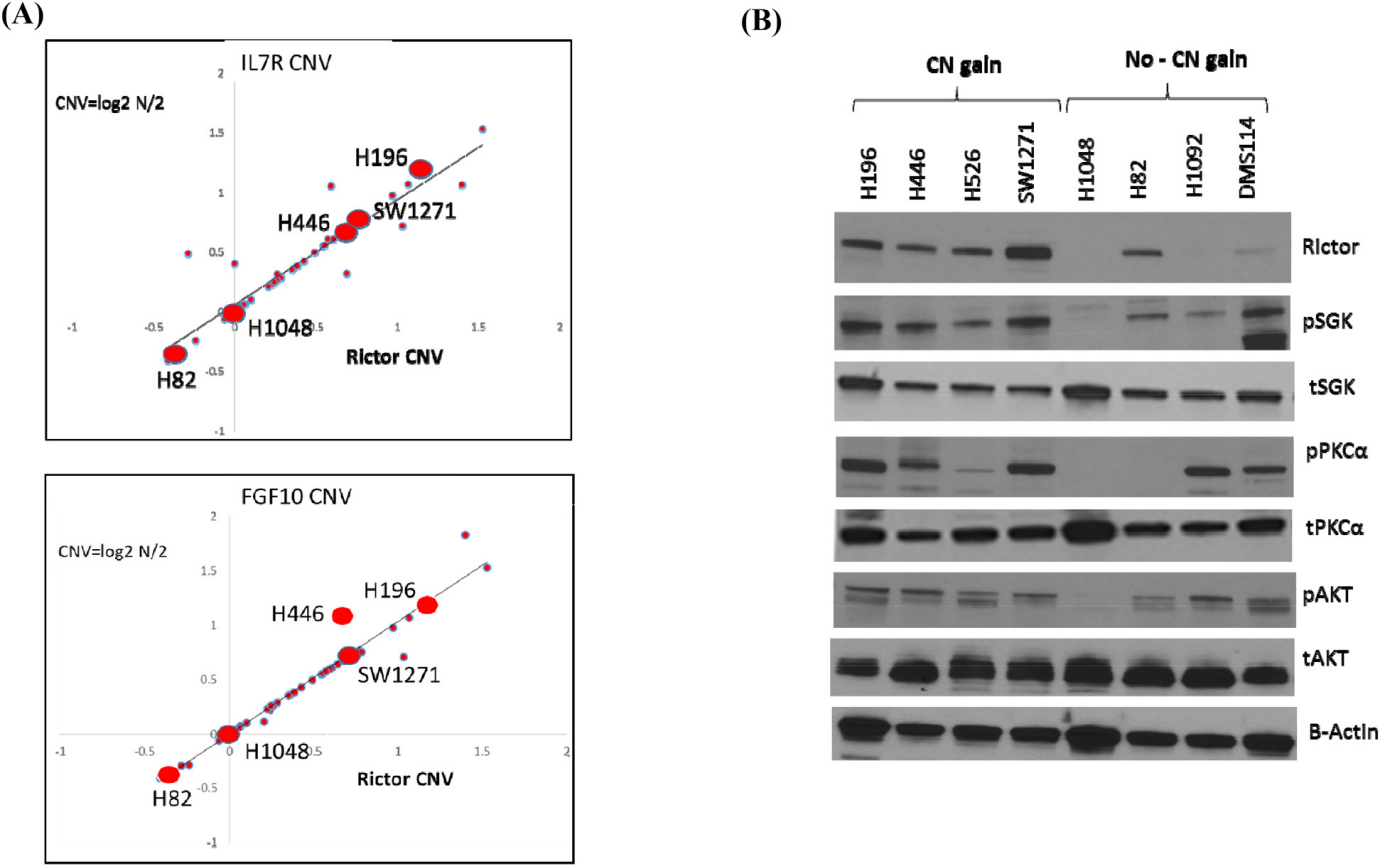
RICTOR expression in SCLC cell lines and tumors **A**. Plots showing Pearson correlation of *RICTOR* with *IL7R* and *FGF10* CNV (p < 0.0001) in SCLC cell lines. Five cell lines used extensively in this study, H196, H446, SW1271, H1048 and H82, are highlighted. **B**. Western blots showing basal expression of RICTOR and its downstream-phosphorylated targets in SCLC cell lines with *RICTOR* CNV. The results show that *RICTOR* CN gain correlates with RICTOR protein expression in SCLC cell lines.

We initially assessed if *RICTOR* CNV paralleled that of *IL7R* and *FGF10* in SCLC cell lines, using data from the Broad Cancer Cell Line Encyclopedia (CCLE) database (http://www.broadinstitute.org/ccle/home) [12]. Indeed, we found parallel CNV among all three genes on chromosome 5p13 (Figure 2A). This suggested that SCLC cell lines are useful model systems to study *RICTOR* amplification.

While *RICTOR* amplification was defined as CNV ≥ 6 for the patient tumor specimens (Figure 1A), among the SCLC cell lines we studied exhibiting a high *RICTOR* CN was H196, with a listed segment mean of 1.1778 (CN~ 4.52). Three other SCLC cell lines we examined with high *RICTOR* CN were H446 (mean 0.6897, CN~ 3.23), H526 (mean 0.6816, CN~ 3.21) and SW1271 (mean 0.7376, CN~ 3.34). Thus, we will refer to all these cell lines as demonstrating *RICTOR* CN gain, rather than amplification. Four additional SCLC cell lines we examined, H1048 (mean -0.0075, CN~ 1.99), H82 (mean -0.3929, CN~ 1.52), H1092 (mean 0.0472, CN~ 2.07) and DMS114 (mean 0.0639, CN~ 2.09) were used as controls because they demonstrated no *RICTOR* CN gain.

Western blotting of SCLC protein lysates demonstrated that a gain in *RICTOR* CN largely correlated with increased RICTOR protein expression (Figure 2B). It was less evident, however, that SCLC cells with *RICTOR* CN gain demonstrated parallel increases in basal mTORC2 complex (RICTOR) signaling, measured by increased phosphorylation of its downstream targets SGK, PKCα, and AKT, compared to cell lines with no gain in *RICTOR* CN (Figure 2B). Thus, in subsequent experiments we focused our studies on SCLC cell lines where downstream signaling best paralleled *RICTOR* CNV: H196, H446, and SW1271 to represent CNV gain, and H1048 and H82 to represent no CNV gain. These cells are highlighted in Figure 2A.

### SCLC cell lines with *RICTOR* CN gain are more sensitive to mTOR inhibitors

**Figure 3:**
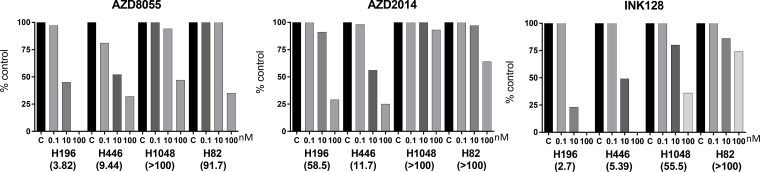
Cell growth inhibition to mTORC inhibitors in SCLC cell lines with RICTOR CNV The effect of three concentrations of mTORC inhibitors (AZD8055, AZD2014, INK128) on cell proliferation was measured by live cell imaging of RFP-labeled cells after 72 h of incubation. Drug was not refreshed during the incubation. Two SCLC cell lines with *RICTOR* CN gain (H446, H196) and two with no gain in *RICTOR* CN (H1048, H82) were studied. Drug doses are listed below bars in nM. The calculated IC50 values, in nM, are listed below the cell names. Cells with *RICTOR* CN gain demonstrate greater sensitivity to mTORC inhibitors.

**Figure 4:**
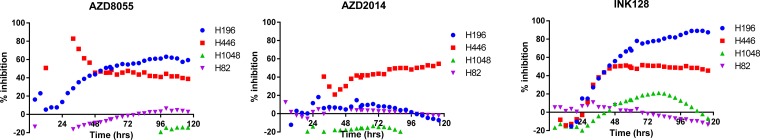
Comparison of growth inhibition produced by a submaximal dose of mTORC inhibitors among SCLC cell lines with RICTOR CNV Cell proliferation data in the presence of 10 nM of mTORC inhibitor (Supplementary Figure 2) was normalized to the respective cell proliferation data in the absence of drug for each time point and collated into a single plot per drug. Some curves appear discontinuous because data points fell outside the plotted range.

We sought to determine whether or not a gain in *RICTOR* CN increases cell sensitivity to drugs targeting the PAM pathway (PI3K/AKT/mTOR). Three different mTOR inhibitors were used in these experiments: AZD8055, AZD2014 and INK128. Initially, the effects of three concentrations of drug (0.1, 10 and 100 nM) on cell growth were examined by measuring total RFP integrated intensity over time (Supplementary Figure 2). All four SCLC cell lines tested (H196, H446, H1048, H82) responded to drug treatment to variable extents; thus IC50s were calculated for each drug and are displayed for individual cell lines (Figure 3). It was clear, however, that cells with high *RICTOR* CNV responded better to all mTOR inhibitors than cells with no *RICTOR* CN gain. Interestingly, AZD2014 demonstrated the most specific, but least potent, inhibitory effect on high vs low *RICTOR* CNV cells. To better demonstrate the response of cells to drug inhibition, we chose to highlight the cell growth response to a submaximal dose (10 nM) of each drug, shown in Figure 4. We found that cell lines with *RICTOR* CN gain (H446 and H196) responded well to 10 nM of all three drugs tested except for the response of H196 to AZD2014. By contrast, cell lines with no *RICTOR* CN gain (H1048 and H82) showed little or no growth inhibition by any of the three drugs when used at this concentration.

**Figure 5:**
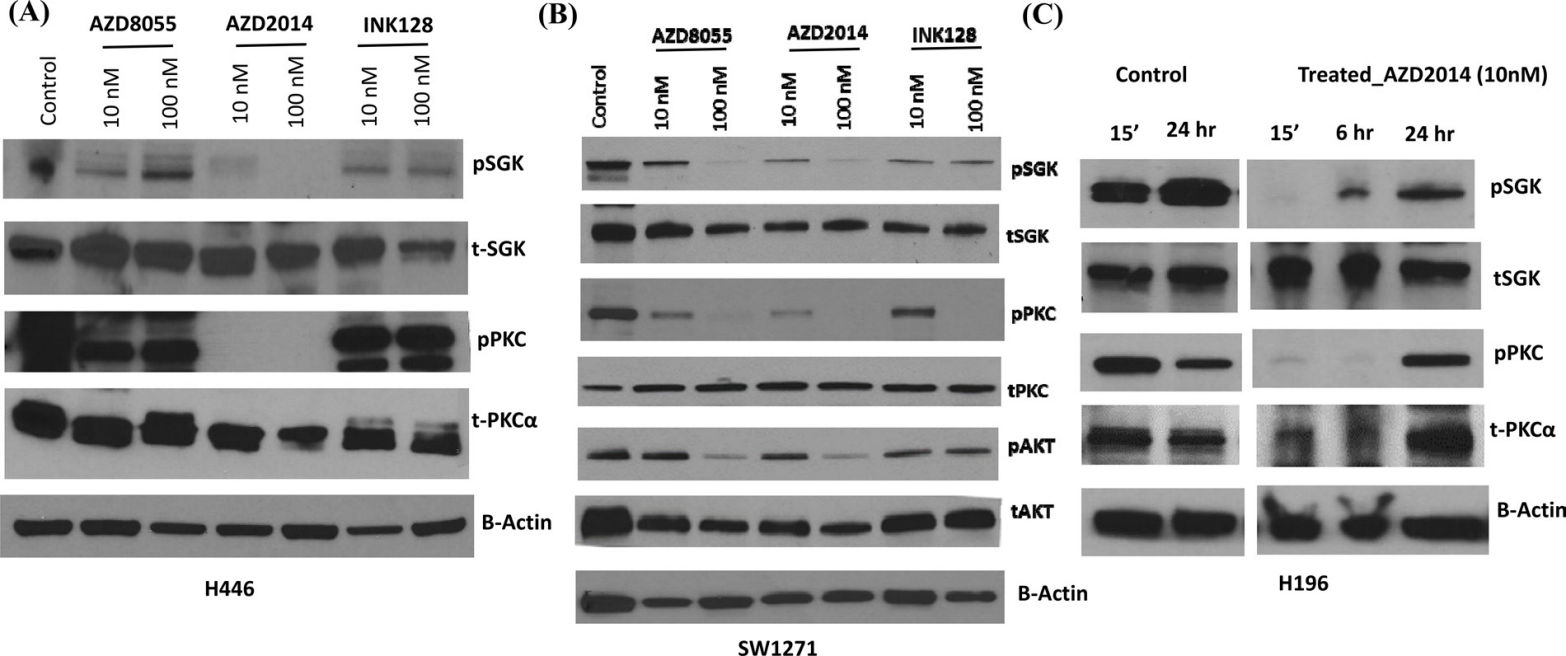
Effect of mTORC inhibitors on downstream signaling in SCLC cells with RICTOR CN gain **A** and **B**. Western blots showing the effect of 15 min incubation of H446 cells (A) and SW1271 cells (B) with mTORC inhibitors (10 and 100 nM) on the phosphorylation of pSGK (Ser-422), pPKC α (Thr-638) and pAKT (Ser-473). **C**. Similar to panels A and B except a time course of H196 cells treated with AZD2014 at 10 nM. β-actin is a loading control for all of these experiments. These results show the greater efficacy of AZD2014 to inhibit downstream mTORC2 signaling in cells with *RICTOR* CN gain.

We next sought to determine the efficacy of these three mTOR inhibitors to decrease the phosphorylation of downstream mTORC2 targets. Using 10 and 100 nM drug concentrations for 15 minutes, AZD2014 almost completely decreased SGK Ser^422^ and PKCα Thr^638^ phosphorylation in H446 cells compared to that produced by AZD8055 and INK128 (Figure 5A). When a second cell line with high *RICTOR* CNV was similarly tested, SW1271, AZD2014 again demonstrated better efficacy than AZD8055, and both were much better than INK128 (Figure 5B). We then treated a third cell line with *RICTOR* CN gain, H196, with AZD2014 for prolonged incubation times using 10 nM drug. We observed that AZD2014 decreased the phosphorylation of downstream SGK and PKCα target proteins for at least 6 hours, but then phosphorylation returned after 24 hours of drug treatment (Figure 5C). Taken together, these results suggest that AZD2014 demonstrates the most consistent inhibition of mTORC2 activity among the three inhibitors tested.

### *RICTOR* CN gain is associated with increased cell motility

**Figure 6:**
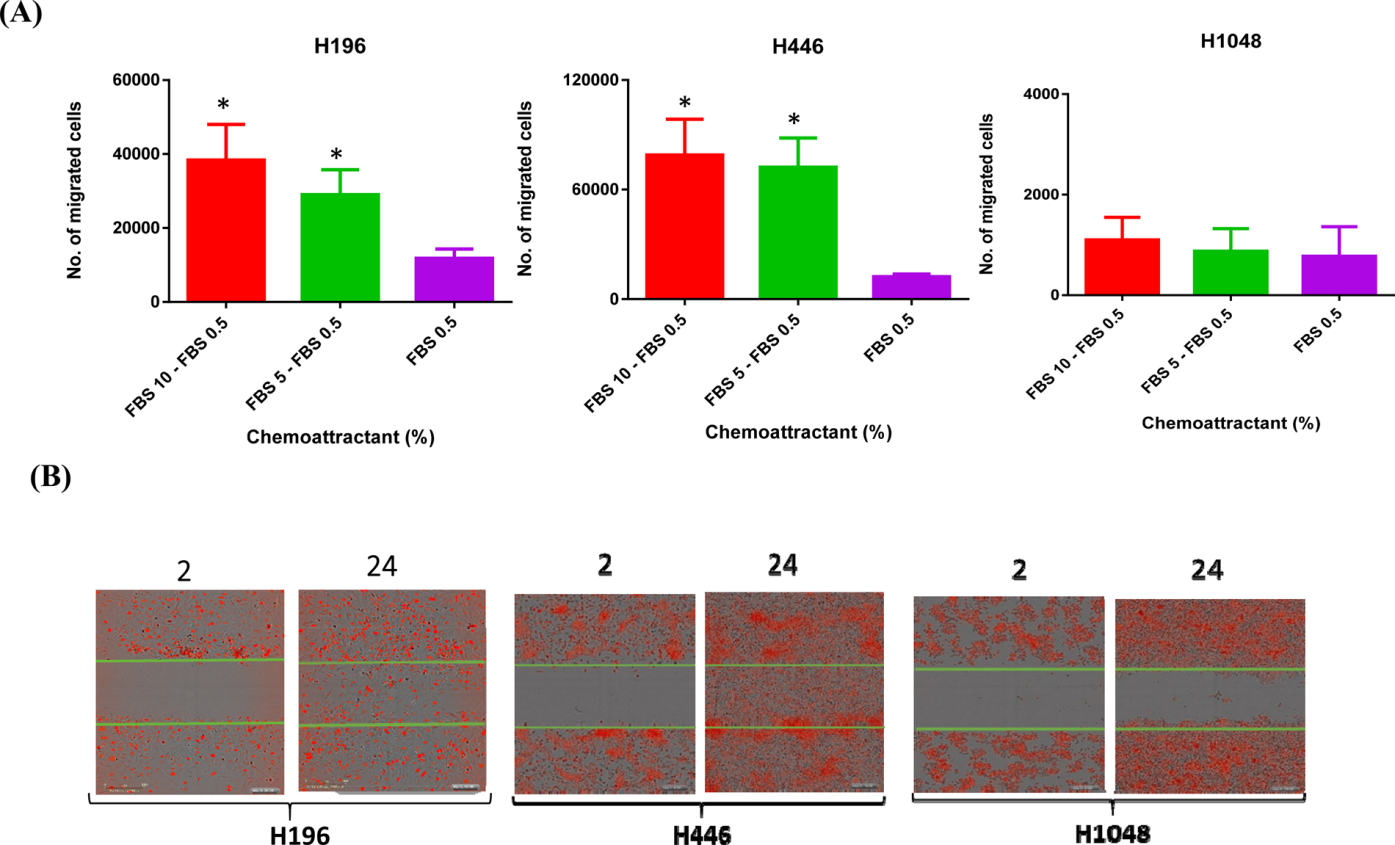
Role of RICTOR CNV in cell migration **A**. Matrigel invasion assay in SCLC cell lines with *RICTOR* CNV. The concentration of chemoattractant is shown on the x-axis, the y-axis shows the number of migrated cells * denotes a significantly higher number of migrated H196 (p <0.0001) and H446 (p <0.0001) cells. Error bars indicate s.d. of means of three independent biological replicates performed in triplicate. **B**. Scratch wound migration assay. Representative images are shown for the indicated time points (hr). Cells with *RICTOR* CN gain demonstrate rapid wound healing compared to cells with no *RICTOR* CN gain.

**Figure 7:**
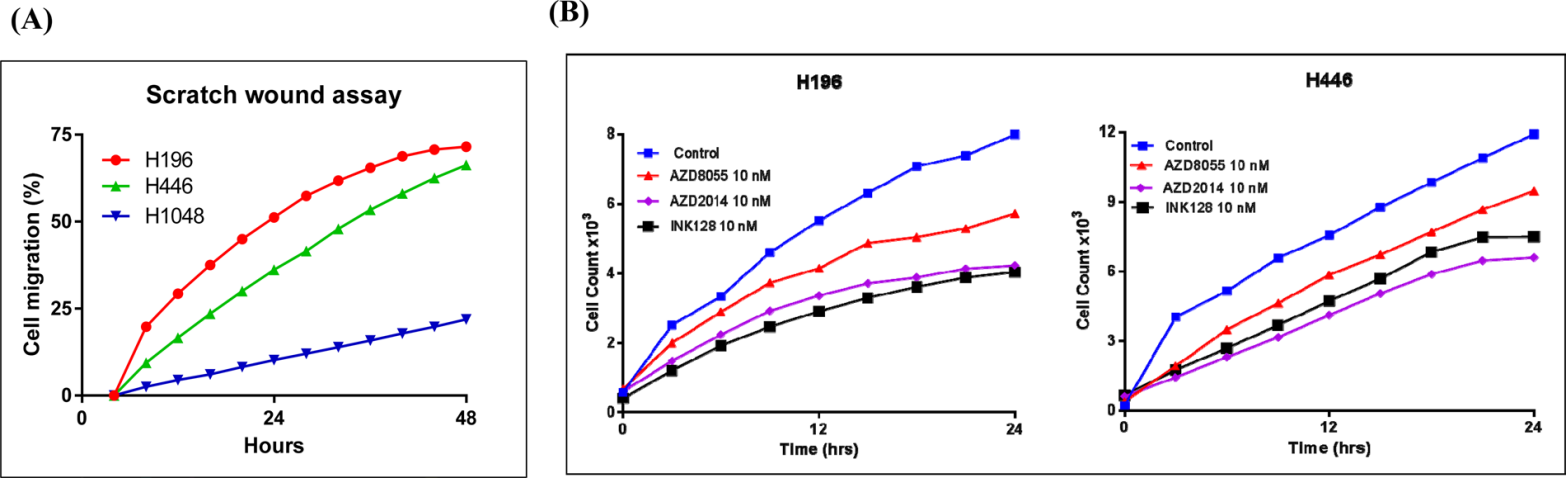
Effect of mTORC inhibitors on cell motility in SCLC cells with RICTOR CN gain **A**. Plot quantitating the migration of cells in a scratch would assay at various times is shown. **B**. Representative results of scratch wound assays showing the effects of mTORC inhibitors (10 nM) on the migration of H196 and H446 cells. Cells with *RICTOR* CN gain demonstrate sensitivity to AZD2014 = INK128 > AZD8055.

Previous studies indicate that *RICTOR* amplification is associated with increased cell motility [9]. Thus, we used two different assays to measure cell motility in SCLC cell lines with *RICTOR* CNV; a directional, chemotactic-induced migration assay and a random, scratch wound migration assay. In chemotaxis assays, significantly greater numbers of cells migrated towards the chemo-attractant (5-10% FBS) using cell lines with *RICTOR* CN gain (H196, H446) compared to a cell line that did not (H1048) (Figure 6A). Similarly, as shown in Figure 6B, the H196 and H446 cell lines closed a scratch wound faster when compared to the H1048 cell line. The conclusion from both migration assays is that cells with *RICTOR* CN gain migrate more rapidly compared to cells with no gain in *RICTOR* CN. A quantitation of the migration plot is shown in the Figure 7A. When the three-mTOR drugs were used at a submaximal dose (10 nM) to assess their effects on cell migration, we observed that cells with *RICTOR* CN gain were more sensitive to INK128 and AZD2014 compared to AZD8055 (Figure 7B).

### *RICTOR* amplification is associated with decreased survival

**Figure 8:**
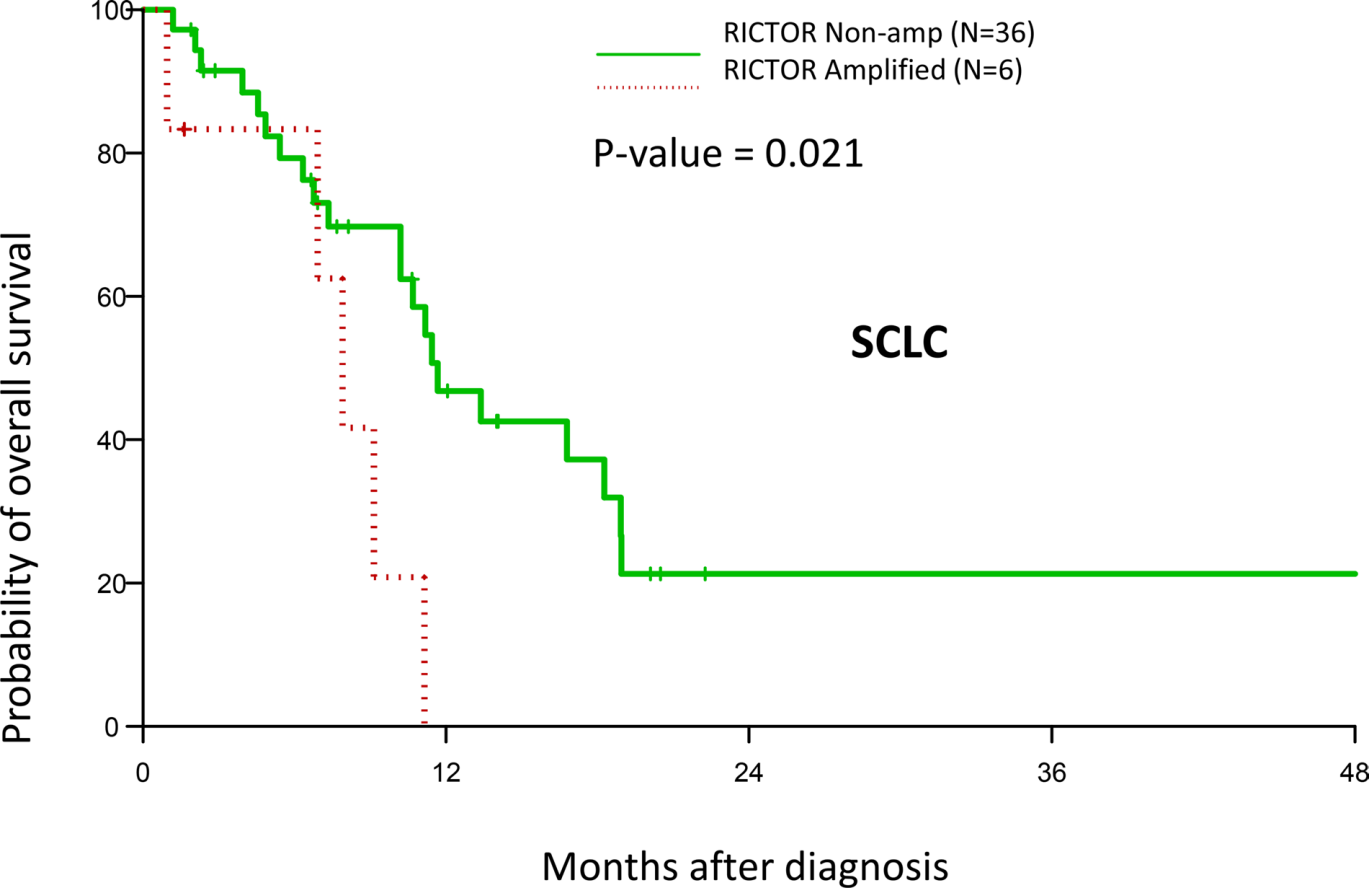
Effect RICTOR amplification on clinical outcome Kaplan–Meier plot on overall survival in SCLC patients with/without *RICTOR* amplification. Patients with *RICTOR* amplification demonstrated reduced survival.

Finally, we sought to determine if *RICTOR* amplification status correlated with clinical outcome in our SCLC cohort. Indeed, we found a significant decrease in overall survival in patients with *RICTOR* amplification (*p* = 0.021) (Figure 8). The median overall survival for *RICTOR* non-amplified and amplified groups were 11.7 months (95% CI: 10.2 - 18.9) and 7.9 months (95% CI: 1 – 11.1), respectively. The detailed clinico-pathological characteristics of SCLC patients, such as age, stage, race, gender and surgery in our cohort is depicted in Table 1.

**Table 1:** Descriptive statistics of SCLC cohort

FACTOR	FREQUENCY (N) = 42
**AGE** (Median 66) years	**Ranges**: 35-85
**Follow up** (Median8.6) months	**Ranges**: 1-55.5
**DISEASE**	
Extensive	33
Limited	09
**RACE**	
White	12
Black	08
Unknown	22
**SURGERY**	
With surgery	01
No surgery	41
**Gender**	
Male	22
Female	20

## DISCUSSION

Copy number variation is a common mechanism by which the expression levels of genes that contribute to cancer development are regulated [13, 14], and contributes to the development of gastric cancer [15], ovarian cancer [16], hepatocellular carcinoma [17], testicular germ cell tumors [18], colorectal carcinoma [19], and bladder cancer [20]. Copies of apoptosis effector genes are often lost during cancer development, in contrast with the frequent amplification of proliferation-related genes [21]. Thus, CNV may represent a mechanism by which transforming cells gain evolutionary advantage. Some established or potential anticancer drug target genes exhibit high levels of CNV, including *HER2* (ERBB2, erb-b2 receptor tyrosine kinase), *TUBB3* (tubulin beta 3 class III) and *TOP2A* (DNA topoisomerase (DNA) II alpha) [22]. Given its value to predict potential deregulation of signaling pathways, CNV may provide useful information for molecular subtyping of SCLC and identification of novel therapeutic strategies.

Here we obtained CNV from targeted exome sequencing of 42 SCLC patients. Three genes (*RICTOR*, *FGF10* and *IL7R*) on chromosome 5p13 were found to be the most frequently amplified in our cohort (Figure 1A). The fact that a fourth gene on chromosome 5p, *SDHA*, was not amplified to a similar extent indicates that there was not a global amplification of this arm of the chromosome. Likewise, there was no parallel amplification of four genes located on chromosome 5q. We focused on *RICTOR* amplification, however, because it represents a potentially actionable gene. The frequency of *RICTOR* amplification observed in the present study, 14%, was slightly higher when compared with previous reports by Ross et al (10%) and Umemura et al (9%) in SCLC [23, 24].

Tumors with *RICTOR* amplification may be sensitive to inhibitors of mTORC2, the RICTOR-containing signaling complex [25]. Numerous inhibitors that target both mTORC1 and mTORC2 complexes, as well as dual PI3K/mTOR inhibitors, are under preclinical and clinical investigation in multiple tumor types [7, 26]. Here, we used the mTORC1/2 inhibitors AZD8055, AZD2014 and INK128 because of their increased potential to inhibit mTORC2 protein synthesis compared to rapalogs [7]. In this regard, there is evidence for both AZD2014 and AZD8055 being potent inhibitors of the mTORCl/2 pathway in breast cancer [27] and lymphoid malignancies [28], and a recent publication also showed efficacy of AZD2014 on NSCLC cell lines [29]. Our data showed that AZD2014 produced the most specific inhibition of growth and downstream signaling among SCLC cell lines with *RICTOR* CNV gain (Figures 3 and 5). Thus, because SCLC cell lines with *RICTOR* CN gain demonstrate sensitivity to mTORC1/2 inhibitors, several of which are currently in clinical trials [30], these drugs may prove effective in SCLC patients harboring tumors with *RICTOR* amplification. Indeed, it was recently reported that an index patient with NSCLC demonstrating *RICTOR*-amplification showed a durable response to the dual mTORC1/2 inhibitor CC-223 [29]. This study also showed that RICTOR over-expression transformed Ba/F3 cells and led to increased sensitivity to mTORC1/2 inhibitors. Thus, *RICTOR* amplification may provide the first predictive biomarker in SCLC to allow enrollment of specific patients into mTOR-directed clinical trials. This would likely improve the initial disappointing results of two mTOR-directed drugs, temsirolimus and everolimus, which failed to show anti-tumor activity in clinical trials of relapsed SCLC [31].

Interestingly, cells with *RICTOR* CN gain also showed increased cell motility (Figures 6A and 6B). A study by Gulhati et al provided evidence of mTORC1/2 regulating epithelial–mesenchymal transition (EMT), motility, and metastasis in colorectal cancer [9]. Furthermore, Gupta et al and others demonstrated a role for mTORC2 specifically to positively regulate TGFβ signaling in cancer cell migration and invasion [8–10, 32, 33]. Thus, *RICTOR* amplification likely promotes increased cell motility *in vitro* via increased mTORC1/2 signaling. This idea is supported by our data that showed that the mTOR inhibitors decreased cell motility in SCLC cells with *RICTOR* CN gain (Figure 7A and 7B). There is similar evidence that mTORC pathway inhibition attenuates migration and invasion of gall bladder cancer via EMT inhibition [33].

We also found a difference in the effect of *RICTOR* amplification on overall survival in SCLC; *RICTOR* amplification was associated with significant decreased survival (*p*=0.021), (Figure 8). The cohort of patients studied was only 42 patients, so it will be of interest to see if the significant effect of *RICTOR* amplification on overall survival is maintained as our cohort grows. In this regard, we have observed that co-amplification of *RICTOR* with *FGF10* and *IL7R* is maintained at a frequency of 13.7% in our latest update of SCLC genomic data using a cohort of N=73 patient tumors (unpublished data). Unfortunately we cannot include data on survival analysis, as this is not yet available.

Taken together, our data suggests that a clinically important subgroup of patients exists in SCLC defined by *RICTOR* amplification. As SCLC is well known as a disease of early and widespread metastasis, it is intriguing to speculate that *RICTOR* amplification may enhance the metastatic process in SCLC by increasing cell motility and in this way affect survival outcome. This idea is supported by immunohistochemical studies showing poor outcome in SCLC patients with tumors demonstrating a mesenchymal phenotype [34, 35]. Similar studies by Li et al using multivariate analysis indicated that TGF-β 1 expression predicted poor survival in SCLC patients. [36].

In conclusion, our study demonstrates the translational potential of mTOR1/2 inhibition as a therapeutic strategy in a subset of SCLC patients with *RICTOR* amplification, which may represent the beginning of a new era of personalized medicine for this disease.

## MATERIALS AND METHODS

### Genomic analysis

Genomic data was obtained from patients who were diagnosed with SCLC at University Hospitals Seidman Cancer Center and had adequate tumor tissue at diagnosis for DNA sequencing [6]. This constituted only 42 of ~500 total SCLC patients currently annotated in our database. A targeted exome sequencing platform is routinely used for genomic analysis of all thoracic tumors and interrogates a panel of 324 clinically relevant cancer-related genes [11]. The platform only makes CNV calls on these 324 targeted genes based upon accumulated sequence coverage for each interrogated exon in the tumor samples compared to a diploid normal control [11]. The typical median coverage for exons is 500x. Genes are considered amplified if the CNV ≥ 6. The specific requirements of sample processing are listed in reference 11. Institutional Review Board approval was obtained to maintain a database of the clinical, molecular and pathological characteristics of all thoracic cancer patients treated at our medical center.

### Cell Lines

All cell lines were purchased within the last three years from ATCC, and have not been authenticated. Cells were maintained as recommended by the supplier. To generate stable cell lines expressing red fluorescent protein (RFP), CellPlayer NucLight Red lentivirus was purchased (Essen Bioscience) and transduced into wild type cells using an MOI of 3. After 48 h, 0.5 μg/ml puromycin was added for selection.

### Western blotting

Protein lysates (40 μg) were analyzed as described previously [37] using 4-20% Criterion gels (Bio-Rad). Gels were transferred in TRIS-glycine running buffer. Antibodies were purchased from: Cell Signaling (RICTOR #2114, pSGK #5599, tSGK #12103, pPKCα #9375, tPKCα #2056, tAKT #9272), Santa Cruz Biotechnology (pAKT #7985) and Sigma (Actin, A-5441).

### Drug treatment

5,000 RFP transduced cells were treated with multiple concentrations of AZD2014, AZD8055 or INK128 (Selleck) in duplicate and allowed to grow for up to 120 h in the IncuCyte Zoom (Essen Bioscience) with fluorescent scanning every 4 h, as described in our previous publication [37] Growth curves were generated showing total RFP integrated intensity over time. The exact chemical structures and Ki of the three mTOR inhibitors can be found at http://www.selleckchem.com/pathways_mTOR.html

### Scratch wound assay

Cells were plated at 35,000 cells per well in Image Lock 96 well plates (Essen BioScience) in 100 μL complete DMEM medium. After overnight incubation, a scratch was made in the plate using a 96-pin WoundMaker tool (Essen BioScience). Plates were washed and incubated for 1 h, and then appropriately diluted drugs were added in 100 μL media to achieve final concentrations. Plates were scanned with an Incucyte Zoom (Essen BioSciences) according to manufacturer's instructions at 2-hour intervals. Images were automatically acquired and registered by the IncuCyte™ software system (ESSEN BioScience). Typically, kinetic updates were recorded over a 24 h period.

### Chemotaxis assay

Invasion assays were performed using the invasion chamber (Essen Biosciences, USA) according to the manufacturer's protocol. Cells were harvested, seeded onto the upper chamber of a Transwell filter that was pre-coated with 10 μg/mL Matrigel, and incubated in RPMI containing 0.5% FBS. After a 24-h incubation at 37°C in 5% CO2, the number of cells that had migrated to the lower chamber containing 5-10% FBS in RPMI media was determined, as described for the scratch wound assay. Control was no FBS gradient, where 0.5% FBS was in the upper and lower chamber. Where indicated, appropriately diluted drugs were added to the upper chamber medium simultaneously with the cells.

### Statistical analysis

Overall survival was measured from the date of diagnosis to the date of death and censored at the date of last follow-up for survivors. Survivor distribution was estimated using Kaplan-Meier methods and differences in survival between groups was examined by the log-rank test. The difference of an interval measure among groups was tested using analysis of variance (ANOVA) and the association between two continuous measures was estimated by the Pearson correlation coefficient. Bar charts and copy-number variant (CNV) plots were used to visualize the sequencing data. Analyses for cell experiments were performed using GraphPad Prism (GraphPad Software, CA, USA). All tests were two-sided and p-values ≤ 0.05 were considered statistically significant. IC50 values were determined by linear regression analysis.

## SUPPLEMENTARY MATERIALS FIGURES AND TABLES





## References

[1] Govindan R, Page N, Morgensztern D, Read W, Tierney R, Vlahiotis A, Spitznagel EL, Piccirillo J (2006). Changing epidemiology of small-cell lung cancer in the United States over the last 30 years: analysis of the surveillance, epidemiologic, and end results database. J Clin Oncol.

[2] Mamdani H, Induru R, Jalal SI (2015). Novel therapies in small cell lung cancer. Transl Lung Cancer Res.

[3] Rudin CM, Durinck S, Stawiski EW, Poirier JT, Modrusan Z, Shames DS, Bergbower EA, Guan Y, Shin J, Guillory J, Rivers CS, Foo CK, Bhatt D (2012). Comprehensive genomic analysis identifies SOX2 as a frequently amplified gene in small-cell lung cancer. Nature genetics.

[4] Peifer M, Fernandez-Cuesta L, Sos ML, George J, Seidel D, Kasper LH, Plenker D, Leenders F, Sun R, Zander T, Menon R, Koker M, Dahmen I (2012). Integrative genome analyses identify key somatic driver mutations of small-cell lung cancer. Nature genetics.

[5] George J, Lim JS, Jang SJ, Cun Y, Ozretic L, Kong G, Leenders F, Lu X, Fernandez-Cuesta L, Bosco G, Muller C, Dahmen I, Jahchan (2015). Comprehensive genomic profiles of small cell lung cancer. Nature.

[6] Dowlati A, Lipka MB, McColl K, Dabir S, Behtaj M, Kresak A, Miron A, Yang M, Sharma N, Fu P, Wildey G (2016). Clinical correlation of extensive-stage small-cell lung cancer genomics. Annals of oncology.

[7] Wander SA, Hennessy BT, Slingerland JM (2011). Next-generation mTOR inhibitors in clinical oncology: how pathway complexity informs therapeutic strategy. The Journal of clinical investigation.

[8] Lamouille S, Connolly E, Smyth JW, Akhurst RJ, Derynck R (2012). TGF-beta-induced activation of mTOR complex 2 drives epithelial-mesenchymal transition and cell invasion. J Cell Sci.

[9] Gulhati P, Bowen KA, Liu J, Stevens PD, Rychahou PG, Chen M, Lee EY, Weiss HL, O'Connor KL, Gao T, Evers BM (2011). mTORC1 and mTORC2 regulate EMT, motility, and metastasis of colorectal cancer via RhoA and Rac1 signaling pathways. Cancer research.

[10] Li H, Lin J, Wang X, Yao G, Wang L, Zheng H, Yang C, Jia C, Liu A, Bai X (2012). Targeting of mTORC2 prevents cell migration and promotes apoptosis in breast cancer. Breast Cancer Res Treat.

[11] Frampton GM, Fichtenholtz A, Otto GA, Wang K, Downing SR, He J, Schnall-Levin M, White J, Sanford EM, An P, Sun J, Juhn F, Brennan K (2013). Development and validation of a clinical cancer genomic profiling test based on massively parallel DNA sequnecing. Nature biotechnol.

[12] Barretina J, Caponigro G, Stransky N, Venkatesan K, Margolin AA, Kim S, Wilson CJ, Lehar J, Kryukov GV, Sonkin D, Reddy A, Liu M, Murray L (2012). The Cancer Cell Line Encyclopedia enables predictive modelling of anticancer drug sensitivity. Nature.

[13] Medina PP, Castillo SD, Blanco S, Sanz-Garcia M, Largo C, Alvarez S, Yokota J, Gonzalez-Neira A, Benitez J, Clevers HC, Cigudosa JC, Lazo PA, Sanchez-Cespedes M (2009). The SRY-HMG box gene, SOX4, is a target of gene amplification at chromosome 6p in lung cancer. Human molecular genetics.

[14] Bowcock AM (2014). Invited review DNA copy number changes as diagnostic tools for lung cancer. Thorax.

[15] Leary RJ, Lin JC, Cummins J, Boca S, Wood LD, Parsons DW, Jones S, Sjoblom T, Park BH, Parsons R, Willis J, Dawson D, Willson JK Integrated analysis of homozygous deletions, focal amplifications, and sequence alterations in breast and colorectal cancers. Proceedings of the National Academy of Sciences of the United States of America.

[16] Despierre E, Moisse M, Yesilyurt B, Sehouli J, Braicu I, Mahner S, Castillo-Tong DC, Zeillinger R, Lambrechts S, Leunen K, Amant F, Moerman P, Lambrechts D (2014). Somatic copy number alterations predict response to platinum therapy in epithelial ovarian cancer. Gynecologic oncology.

[17] Xu H, Zhu X, Xu Z, Hu Y, Bo S, Xing T, Zhu K (2015). Non-invasive Analysis of Genomic Copy Number Variation in Patients with Hepatocellular Carcinoma by Next Generation DNA Sequencing. Journal of Cancer.

[18] Silveira SM, da Cunha IW, Marchi FA, Busso AF, Lopes A, Rogatto SR (2014). Genomic screening of testicular germ cell tumors from monozygotic twins. Orphanet journal of rare diseases.

[19] Horpaopan S, Spier I, Zink AM, Altmuller J, Holzapfel S, Laner A, Vogt S, Uhlhaas S, Heilmann S, Stienen D, Pasternack SM, Keppler K, Adam R (2015). Genome-wide CNV analysis in 221 unrelated patients and targeted high-throughput sequencing reveal novel causative candidate genes for colorectal adenomatous polyposis. International journal of cancer.

[20] Bonberg N, Pesch B, Behrens T, Johnen G, Taeger D, Gawrych K, Schwentner C, Wellhausser H, Kluckert M, Leng G, Nasterlack M, Oberlinner C, Stenzl A (2014). Chromosomal alterations in exfoliated urothelial cells from bladder cancer cases and healthy men: a prospective screening study. BMC cancer.

[21] Mauro JA, Butler SN, Ramsamooj M, Blanck G (2015). Copy number loss or silencing of apoptosis-effector genes in cancer. Gene.

[22] Labots M, Buffart TE, Haan JC, van Grieken NC, Tijssen M, van de Velde CJ, Grabsch HI, Ylstra B, Carvalho B, Fijneman RJ, Verheul HM, Meijer GA (2014). High-level copy number gains of established and potential drug target genes in gastric cancer as a lead for treatment development and selection. Cellular oncology.

[23] Ross JS, Wang K, Elkadi OR, Tarasen A, Foulke L, Sheehan CE, Otto GA, Palmer G, Yelensky R, Lipson D, Chmielecki J, Ali SM, Elvin J (2014). Next-generation sequencing reveals frequent consistent genomic alterations in small cell undifferentiated lung cancer. J Clin Pathol.

[24] Umemura S, Mimaki S, Makinoshima H, Tada S, Ishii G, Ohmatsu H, Niho S, Yoh K, Matsumoto S, Takahashi A, Morise M, Nakamura Y, Ochiai A (2014). Therapeutic priority of the PI3K/AKT/mTOR pathway in small cell lung cancers as revealed by a comprehensive genomic analysis. J Thorac Oncol.

[25] Sparks CA, Guertin DA (2010). Targeting mTOR: prospects for mTOR complex 2 inhibitors in cancer therapy. Oncogene.

[26] Schenone S, Brullo C, Musumeci F, Radi M, Botta M (2011). ATP-competitive inhibitors of mTOR: an update. Current medicinal chemistry.

[27] Leung EY, Askarian-Amiri M, Finlay GJ, Rewcastle GW, Baguley BC (2015). Potentiation of Growth Inhibitory Responses of the mTOR Inhibitor Everolimus by Dual mTORC1/2 Inhibitors in Cultured Breast Cancer Cell Lines. PloS one.

[28] Eyre TA, Collins GP, Goldstone AH, Cwynarski K (2014). Time now to TORC the TORC? New developments in mTOR pathway inhibition in lymphoid malignancies. British journal of haematology.

[29] Cheng H, Zou Y, Ross JS, Wang K, Liu X, Halmos B, Ali SM, Liu H, Verma A, Montagna C, Chachoua A, Goel S, Schwartz EL (2015). RICTOR Amplification Defines a Novel Subset of Patients with Lung Cancer Who May Benefit from Treatment with mTORC1/2 Inhibitors. Cancer discovery.

[30] Pike KG, Malagu K, Hummersone MG, Menear KA, Duggan HM, Gomez S, Martin NM, Ruston L, Pass SL, Pass M (2013). Optimization of potent and selective dual mTORC1 and mTORC2 inhibitors: the discovery of AZD8055 and AZD2014. Bioorganic & medicinal chemistry letters.

[31] D'Angelo SP, Pietanza MC (2010). The molecular pathogenesis of small cell lung cancer. Cancer biology & therapy.

[32] Gupta S, Hau AM, Al-Ahmadie HA, Harwalkar J, Shoskes AC, Elson P, Beach JR, Hussey GS, Schiemann WP, Egelhoff TT, Howe PH, Hansel DE (2016). Transforming Growth Factor-beta Is an Upstream Regulator of Mammalian Target of Rapamycin Complex 2-Dependent Bladder Cancer Cell Migration and Invasion. The American journal of pathology.

[33] Zong H, Yin B, Zhou H, Cai D, Ma B, Xiang Y (2014). Inhibition of mTOR pathway attenuates migration and invasion of gallbladder cancer via EMT inhibition. Molecular biology reports.

[34] Galvan JA, Astudillo A, Vallina A, Crespo G, Folgueras MV, Gonzalez MV (2014). Prognostic and diagnostic value of epithelial to mesenchymal transition markers in pulmonary neuroendocrine tumors. BMC cancer.

[35] Canadas I, Rojo F, Taus A, Arpi O, Arumi-Uria M, Pijuan L, Menendez S, Zazo S, Domine M, Salido M, Mojal S, Garcia de Herreros A, Rovira A (2014). Targeting epithelial-to-mesenchymal transition with Met inhibitors reverts chemoresistance in small cell lung cancer. Clin Cancer Res.

[36] Li XX, Li RJ, Zhao LJ, Liu NB, Wang P (2015). Expression of molecular factors correlated with metastasis in small cell lung cancer and their significance. Int J Clin Exp Pathol.

[37] Dabir S, Babakoohi S, Kluge A, Morrow JJ, Kresak A, Yang M, MacPherson D, Wildey G, Dowlati A (2014). RET mutation and expression in small-cell lung cancer. J Thorac Oncol.

