# Supplementary material for: RICTOR amplification identifies a subgroup in small cell lung cancer and predicts response to drugs targeting mTOR
Source: Oncotarget. 2016 Nov 15;8(4):5992–6002. doi: 10.18632/oncotarget.13362 (PMC5351607; doi:10.18632/oncotarget.13362)
Supplement: Supplementary file 1 [file oncotarget-08-5992-s001.pdf]

RICTOR amplification identifies a subgroup in small cell lung cancer and predicts response to drugs targeting mTOR

SUPPLEMENTARY FIGURES AND TABLE

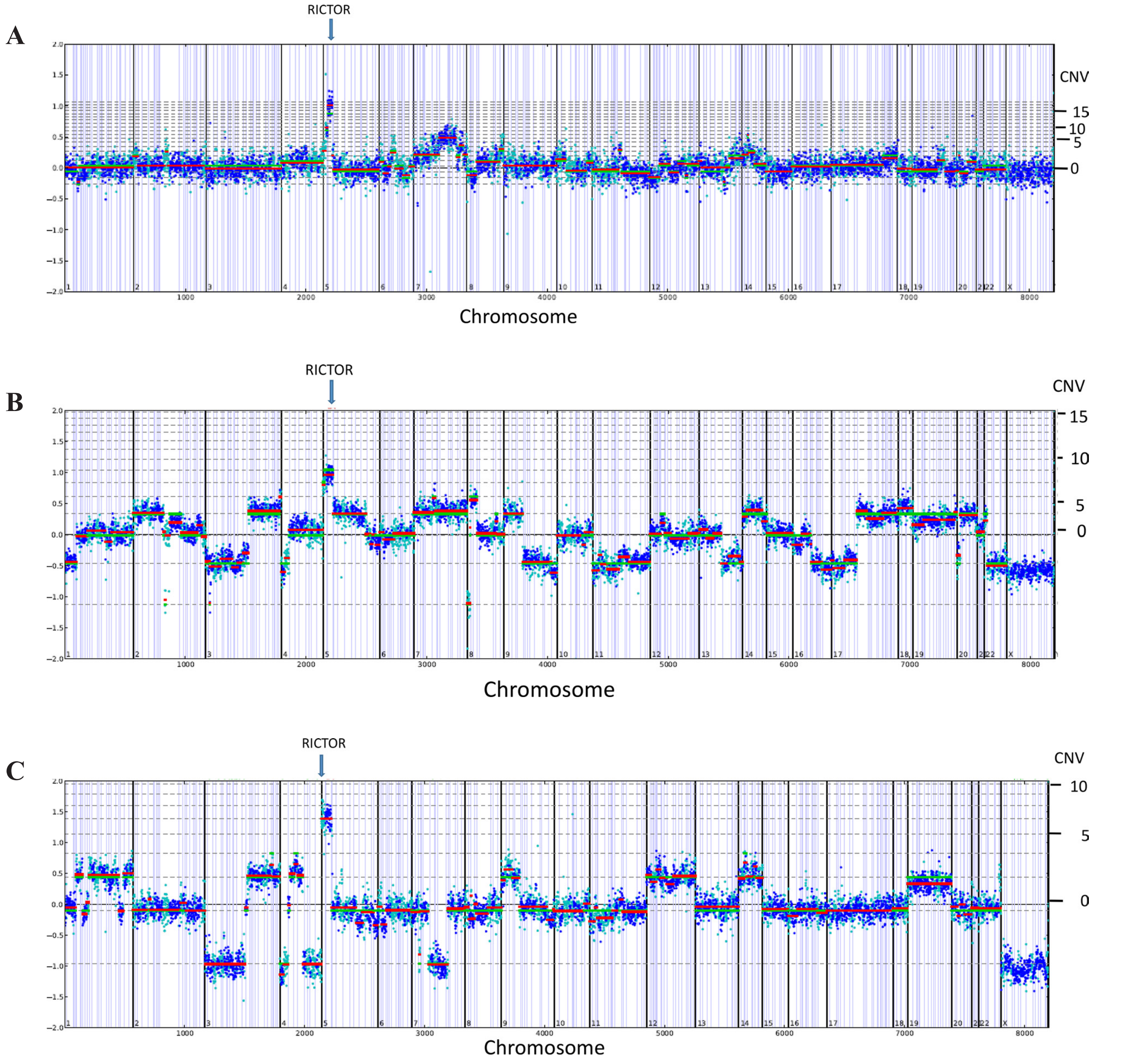

Supplementary Figure 1: A. CNV plot of a SCLC tumor sample with amplification of the *RICTOR* gene at 18 copies on 5p13. The chromosomal location is provided on the X-axis and the gene copy number on the Y-axis. **B.** CNV plot of a SCLC tumor sample with amplification of the *RICTOR* gene at 7 copies on 5p13. The chromosomal location is provided on the X-axis and the gene copy number on the Y-axis. **C.** CNV plot of a SCLC tumor sample with amplification of the *RICTOR* gene at 6 copies on 5p13. The chromosomal location is provided on the X-axis and the gene copy number on the Y-axis.

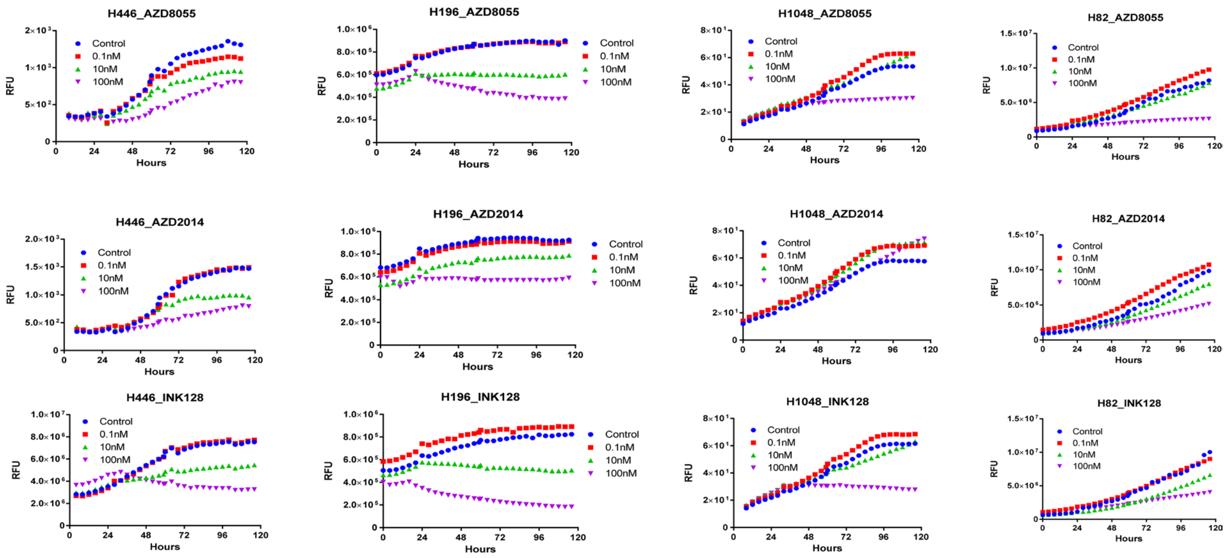

Supplementary Figure 2: The effect of mTORC inhibitors (AZD8055, AZD2014, INK128) on cell proliferation in two SCLC cell lines with *RICTOR* CN gain (H446, H196) and two with no gain in *RICTOR* CN (H1048, H82) were studied.

Supplementary Table: 1: List of genes demonstrating amplification in SCLC patient tumors

See Supplementary File 1.
